# Staged microsurgical resection of supra-/parasellar meningioma with cavernous sinus exenteration and carotid-to–middle cerebral artery bypass

**DOI:** 10.3171/2025.10.FOCVID25163

**Published:** 2026-01-01

**Authors:** Umid Sulaimanov, Franco Vera Figueroa, Abdullah Keles, Ufuk Erginoglu, Yerkebulan Serikkanov, Umut Tan Sevgi, Oyku Ozturk, Mustafa K. Baskaya

**Affiliations:** Department of Neurological Surgery, School of Medicine and Public Health, University of Wisconsin, Madison, Wisconsin

**Keywords:** anterior skull base, suprasellar meningioma, cerebral bypass, microsurgery, extracranial-intracranial bypass, skull base tumor

## Abstract

Atypical skull base meningiomas are characterized by aggressive growth and neurovascular invasion, which complicate resection and predispose to high recurrence rates. Lesions encasing major vessels and cranial nerves or refractory to surgery and radiation therapy are associated with significant morbidity, while systemic therapies remain largely ineffective despite ongoing trials. Their management, therefore, necessitates advanced microsurgical strategies. We describe a radiation-induced WHO grade II atypical meningioma extensively involving the cavernous sinus, supra-/parasellar, and petroclival regions in a patient with 6 prior surgeries, 2 Gamma Knife treatments, and multiple systemic therapies, presenting with ophthalmoplegia and right-eye blindness, treated using a three-stage surgical approach.

The video can be found here: https://stream.cadmore.media/r10.3171/2025.10.FOCVID25163

## Transcript

We present the case of a radiation-induced supra-/parasellar meningioma extensively involving the orbit, cavernous sinus, and the petroclival region in a patient with multiple prior surgeries, Gamma Knife radiation, chemotherapy, and experimental treatments. The tumor showed progressive growth across the anterior, middle, and posterior cranial fossae with encasement of the internal carotid artery (ICA) and its branches. Management required a staged strategy, involving a common carotid artery (CCA)–to–middle cerebral artery (MCA) bypass using a radial artery graft, followed by partial orbital exenteration and complete cavernous sinus exenteration with supra- and parasellar tumor resection via a pterional recraniotomy. A third retrosigmoid stage addressed the petroclival component, but this video focuses on the first two stages relevant to the anterior skull base.

### 1:02 Background Information.

Meningiomas of the anterior skull base and the middle fossa are optimally approached via a pterional-transsylvian route, allowing safe access to critical structures.^1^ When these tumors encase the ICA and its branches, preoperative planning may require cerebral revascularization to maintain adequate cerebral perfusion if parent artery sacrifice or trapping becomes necessary. Extension into the cavernous sinus, clivus, or petroclival region may necessitate more extensive techniques, such as the Dolenc approach for cavernous sinus involvement and the Kawase approach for the petrous apex.^2,3^ In selected cases, staged microsurgery enables the maximal resection while minimizing morbidity.^4^

### 1:38 First-Stage Surgery.

We present the case of a 48-year-old man with childhood cranial radiation and chemotherapy for histiocytosis, complicated by panhypopituitarism, who developed a progressive WHO grade II meningioma. Despite multiple prior surgeries, Gamma Knife radiation, and systemic therapies, the tumor enlarged, involving the supra- and parasellar regions with extension into the orbit, cavernous sinus, petroclival, and prepontine areas. He presented with complete blindness, ophthalmoplegia, right-sided facial weakness, hearing loss, and sensory loss, and was referred for further management.

### 2:08 Preoperative Imaging.

Magnetic resonance imaging (MRI) revealed a large right-sided skull base tumor, measuring approximately 6 cm, involving the supra- and parasellar regions, orbital apex, cavernous sinus, Meckel’s cave, and petroclival and prepontine areas. The lesion completely encased the ICA from its cavernous segment to the bifurcation of the ICA. Progressive extension toward the optic chiasm and contralateral optic nerve was of particular concern in the setting of right-sided blindness.

### 2:33 Management.

Due to the involvement of critical neurovascular structures, a staged surgical approach was planned. Cerebral angiography with balloon test occlusion under electroencephalography (EEG) and hypotensive challenge demonstrated intolerance to ICA sacrifice, necessitating an extracranial-to-intracranial (EC-IC) bypass in the first stage to maintain adequate cerebral perfusion.^5,6^ At the second stage, temporary ICA occlusion was performed with continuous monitoring of motor evoked potentials (MEPs), somatosensory evoked potentials (SSEPs), and EEG to assess tolerance for additional vessel sacrifice. In areas where occlusion was tolerated, specifically the posterior communicating artery (PComA) and the anterior choroidal artery (AChA), collateral circulation was sufficient to maintain perfusion, permitting safe resection with sacrifice of these branches. Once vascular safety was confirmed, tumor removal proceeded. Any posterior fossa component that could not be safely removed during this stage was addressed with a third-stage retrosigmoid approach.

### 3:24 Patient Positioning, Skin Incision, and Neck Dissection.

Under general anesthesia, the patient was positioned supine with the head turned left. The forearm radial artery was prepared for graft harvest, prior incisions were marked, neuronavigation registered, and cervical exposure performed along the sternocleidomastoid to access the carotid bifurcation. Attention was then directed to the cranial site.

### 3:41 Dural Opening and Arachnoid Dissection.

The prior cranial incision was reopened, the bone flap was elevated, and the dura was incised. The sylvian fissure was carefully dissected despite dense adhesions to expose M2 branches for bypass, with limited tumor resection for pathology. Further removal near the ICA was deferred to the second stage to minimize the risk of vascular complications.

Thereafter, the M2 segment was prepared for end-to-side bypass, and heparin was administered before temporary clipping. A radial artery graft was harvested, adventitia trimmed, and the distal end fishmouthed. Distal anastomosis to M2 was completed with interrupted 8-0 nylon sutures. Proximal anastomosis to the CCA was performed due to the high carotid bifurcation in the neck. After arteriotomy enlargement with an aortic punch, anastomosis was completed with interrupted 8-0 nylon sutures. Graft patency and flow were confirmed with Doppler and indocyanine green (ICG) angiography. Subsequently, to prevent competitive flow, the cervical ICA was ligated just above the bifurcation. Standard wound closure was performed, and the patient was extubated, remaining neurologically stable. A postoperative computed tomography angiogram confirmed bypass patency.

### 5:29 Second-Stage Surgery.

Two days later, the patient underwent the second stage of tumor resection. The pterional craniotomy was reopened, the dura elevated, and bypass patency confirmed with micro-Doppler. The lower abdomen was also prepared for possible fat graft harvest.

The distal sylvian fissure was opened proximal to the bypass, and the MCA bifurcation with M2 branches was reidentified. Dissection was then carried proximally along M1 to locate the ICA bifurcation and A1 segment. The M1 segment was heavily encased, particularly laterally, and careful dissection was required to separate it from the tumor. A cotton pledget was observed at the most proximal supraclinoid ICA, likely placed during a prior surgery for hemostasis. Once freed, the A1 and ICA bifurcations were exposed, and further cleaning of these structures was performed. The ipsilateral optic nerve and chiasm were identified, while the entire supraclinoid ICA was found to be encased. Because a segment was needed for distal trapping, further dissection was attempted and, with difficulty, a 2- to 3-mm segment of ICA just before the bifurcation was secured. Proximal exposure of the ICA, including the PComA and AChA, was not feasible due to severe involvement and scarring.

At this stage, a decision regarding trapping was required, as the PComA and AChA could not be identified preoperatively or during dissection. To assess tolerance, an intraoperative surrogate occlusion test was performed by placing a temporary clip on the ICA just before its bifurcation, with continuous monitoring of MEP, SSEP, and EEG every 5 minutes for 1 hour under mild hypotension. Any neurophysiological change would have indicated patency of the PComA or AChA, precluding safe cavernous sinus exenteration. During this occlusion test, resection of the orbital tumor component was performed, limited to partial exenteration per the patient’s cosmetic preference. After 1 hour of stable monitoring, it was evident that the PComA and AChA were nonfunctional or occluded by the tumor, with collateral circulation supplying their territories.

Following trapping of the ICA segment between the bifurcation clip and the previously ligated cervical ICA, a safe corridor was established for dissection and tumor removal in the middle fossa and the cavernous sinus. Using sharp dissection and the ultrasonic aspirator, the firm tumor was meticulously resected, progressing medially and anteriorly. During cavernous sinus resection, the temporary ICA clip was exchanged for a permanent clip to secure the vessel. As dissection continued, the posterior tumor surface was reached, permitting visualization of the brainstem and basilar artery, and the tumor was removed from the prepontine cistern with care to preserve the pia and basilar perforators. The proximal remnant of the third cranial nerve was identified. Dissection then proceeded posteriorly into the infratemporal compartment, where the tumor was shaved down, leaving only a portion within the cerebellopontine angle and petroclival region for a planned third-stage surgery. Hemostasis was achieved, a fat graft was placed to line the resection cavity, and incisions were closed in standard layered fashion.

### 8:59 Postoperative Imaging and Course.

The surgery was uneventful, and the patient awoke without additional neurological deficits compared to the preoperative baseline. Following the third-stage retrosigmoid resection of the residual tumor, gross-total resection was achieved. Pathology confirmed a WHO grade II atypical meningioma. In the postoperative course, the patient developed communicating hydrocephalus, which was managed with a ventriculoperitoneal shunt, and was ultimately discharged to rehabilitation and then home.

### 9:23 Conclusion.

Staged surgery enables maximal resection of complex skull base tumors while limiting neurovascular risk.^7^ Adjuncts such as neuromonitoring, intraoperative ICG angiography, and neuronavigation provide real-time guidance for vessel exclusion and dissection planes and, when combined with meticulous microsurgery, enhance the likelihood of successful tumor removal and favorable outcomes in challenging skull base cases.^8^

## Disclosures

The authors report no conflict of interest concerning the materials or methods used in this study or the findings specified in this publication.

## Author Contributions

Primary surgeon: Baskaya. Editing and drafting the video and abstract: Sulaimanov, Vera Figueroa, Keles, Erginoglu, Serikkanov, Sevgi, Ozturk. Critically revising the work: Baskaya, Sulaimanov, Keles, Erginoglu, Serikkanov, Sevgi, Ozturk. Reviewed submitted version of the work: all authors. Approved the final version of the work on behalf of all authors: Baskaya. Supervision: Baskaya.

## Supplemental Information

### Patient Informed Consent

The necessary patient informed consent was obtained in this study.
